# Tumor‐derived extracellular vesicles and particles (EVPs): pivotal vectors in driving metabolic reprogramming

**DOI:** 10.1002/mco2.434

**Published:** 2023-11-29

**Authors:** Xinming Su, Peijie Zheng, Shiwei Duan

**Affiliations:** ^1^ Key Laboratory of Novel Targets and Drug Study for Neural Repair of Zhejiang Province School of Medicine, Hangzhou City University Hangzhou Zhejiang China; ^2^ Department of Clinical Medicine Hangzhou City University Hangzhou Zhejiang China

**Keywords:** cancer, chemotherapy toxicity, extracellular vesicles and particles, liver metabolism, metabolic reprogramming, nonalcoholic fatty liver disease

1

A recent research published in *Nature* by Wang et al.^1^ highlights the significant role of tumor‐derived extracellular vesicles and particles (EVPs) in mediating metabolic reprogramming within the liver. The study demonstrates that these EVPs, even in the absence of liver metastasis, contribute to the development of nonalcoholic fatty liver disease (NAFLD) and inflammation, impair the liver's drug metabolism capabilities, and increase the toxicity of chemotherapy drugs to the body.

Dysregulation of cellular metabolism is a prominent hallmark of cancer.^2^ Not only do cancer cells undergo their own metabolic remodeling, but they also exert influence on the metabolism of neighboring or distant cells in the body through various mechanisms, such as metabolite secretion and modulation of receptor expression. Consequently, these alterations impact a range of physiological functions.^3^ EVPs constitute a diverse class of soluble factors with the ability to modify the metabolic microenvironment within target cells as well as in their surrounding milieu.^3^ Moreover, EVPs play a pivotal role as essential mediators for substance and signal transmission during both pathological and physiological states within cells.^4^ Despite advancing insights into EVPs, studies investigating their impact on cellular metabolism remain relatively limited. Specifically, the intricate mechanisms by which EVPs influence the reprogramming of metabolic functions in specific diseases, including cancer and nervous system disorders, have yet to be comprehensively elucidated.

To explore the influence of tumors on liver metabolism, Wang et al. employed a cancer cell line devoid of liver metastasis properties to establish an animal model. They conducted a series of comprehensive multiomics analyses to investigate this phenomenon thoroughly. Strikingly, despite the absence of metastasis to the liver, significant metabolic dysregulation was observed in the nonmetastatic livers compared to normal livers. This dysregulation is characterized by the upregulation of pathways associated with immune homeostasis, such as inflammation and the nonclassical tumor necrosis factor (TNF)‐mediated nuclear factor‐kappa B signaling pathway, which serves as a pivotal connection between inflammation and cancer. Simultaneously, there is a concurrent downregulation of metabolic pathways, including oxidative phosphorylation (the process of synthesizing adenosine triphosphate through phosphorylation of adenosine diphosphate) and fatty acid metabolism. Consequently, lipid and amino acid metabolic pathways are downregulated, leading to the accumulation of diverse lipids within the liver. This finding was further corroborated by experimental results obtained from various cell lines and patient liver biopsies. This intriguing observation suggests the potential transmission of a specific “signal” by tumor cells through a distinct medium, indirectly influencing metabolic changes in the liver.

Moreover, the authors delved into the intricate molecular mechanisms underlying this phenomenon. Initially, they isolated and confirmed that EVPs derived from tumor cells have the capability to be absorbed by the liver, thereby orchestrating metabolic alterations in the organ, including dysregulation of pertinent pathways and the appearance of analogous metabolites. Drawing insights from multiple prior studies, the authors delineated that tumor cells efficiently secrete EVPs via Rab27a, a core protein regulating exosome secretion. Notably, the palmitic acid within these EVPs selectively targets Kupffer cells in the liver. This intricate process triggers toll‐like receptor 4‐dependent secretion of TNF by Kupffer cells, ultimately leading to the onset of NAFLD (Figure 1). Considering the widespread distribution and evasive nature of EVPs from immune clearance, the authors propose and affirm the tremendous clinical translational potential of EVPs as essential mediators enabling tumor‐induced “remote control” over liver metabolism.

**FIGURE 1:**
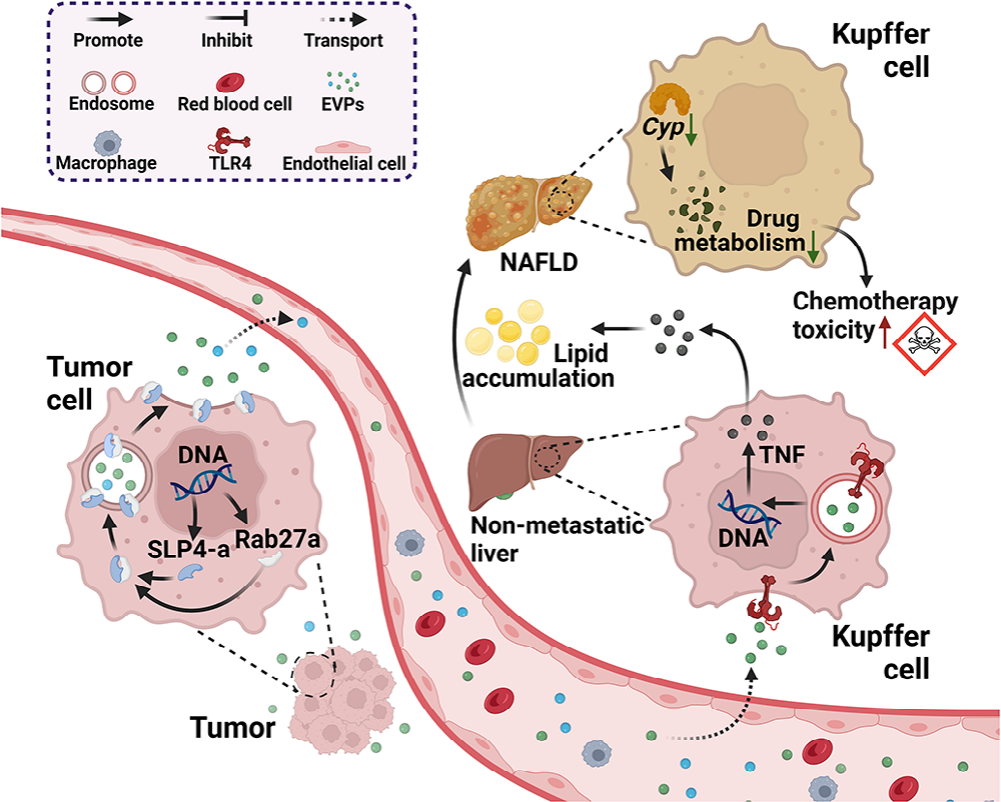
Molecular mechanism of tumor‐derived EVPs in liver metabolic reprogramming carcinoma in situ tumor cells possess the ability to promote the release of endosomes and the generation of EVPs by expressing Rab27a and SLP4‐a, which assemble into complexes on the surface of endosomes. These EVPs can be transported to the liver through the bloodstream and are taken up by Kupffer cells in a TLR4‐dependent manner, subsequently releasing their cargo. One notable component is palmitic acid, which upon cellular entry stimulates the secretion of TNF. This, in turn, leads to lipid droplet accumulation, resulting in fatty liver development. Consequently, the metabolism of Cyp enzymes responsible for processing chemotherapy drugs becomes inhibited, thereby amplifying the toxicity of chemotherapy. The abbreviations Cyp (cytochrome), NAFLD (nonalcoholic fatty liver disease), TNF (tumor necrosis factor), and TLR4 (toll‐like receptor 4) are utilized in this context.

In the final phase of their study, the authors investigated the influence of liver metabolic disorders mediated by tumor‐derived EVPs on drug metabolism and chemotherapy toxicity. This expansion of their research scope enhances the clinical relevance and significance of their findings. Through RNA sequencing and gene set enrichment analysis, the authors identified the downregulation of core genes in hepatocytes, which are the human counterparts of cytochrome P450 enzymes crucial for metabolizing most prescribed drugs. Notably, TNF blockade effectively rescued this impairment, indicating that tumor‐derived EVPs hinder liver drug metabolism via TNF‐related pathways. Additionally, the authors investigated the impact of NAFLD on chemotherapy toxicity and demonstrated that tumor EVPs could enhance the toxicity of chemotherapy drugs such as dacarbazine and doxorubicin (including bone marrow and heart toxicity) by compromising liver drug metabolism, consequently reducing chemotherapy resistance.

The groundbreaking research conducted by Wang and colleagues establishes a crucial connection between carcinoma in situ, EVPs, and liver metabolism. This study not only provides a fresh perspective and foundation for related basic research and clinical treatments but also holds significant potential for clinical translation. Their findings indicate that carcinoma in situ can potentially induce physiological changes in multiple organs, both metastatic and nonmetastatic. Consequently, cancer treatments should systematically consider the impact on the metabolism of systemic organs. Initiatives aimed at enhancing the physiological conditions of these related organs may enhance the efficacy of existing treatments. Additionally, the identification of large, readily available, and specific tumor‐derived EVPs as key elements in inducing metabolic function remodeling opens avenues for early cancer detection, patient classification, treatment efficacy assessment, and potential therapeutic interventions.

However, their research ideas appear somewhat limited, relying heavily on existing studies for their mechanistic understanding. While the authors have identified a signaling pathway involving tumor‐derived EVPs and palmitic acid that mediates liver metabolic reprogramming, the intricate nature of cell metabolism suggests that palmitic acid may simultaneously regulate liver inflammation and contribute to NAFLD formation through multiple metabolic pathways. Whether this pathway represents the primary mechanism by which EVPs influence liver metabolism via palmitic acid remains uncertain. Several related pathways that potentially contribute to NAFLD development have been overlooked and warrant further investigation. For instance, xenobiotic metabolism can convert lipophilic compounds into hydrophilic metabolites, facilitating their elimination and reducing the incidence and progression of NAFLD.^5^ Furthermore, given the diverse range of substances contained in EVPs, even those present in lower concentrations may play critical roles in liver metabolism. Additionally, while the authors have confirmed that EVPs subpopulations isolated from B16F10 cells exhibit varying degrees of inflammation induction and alteration of liver metabolism, they have not delved into the specific mechanisms underlying these EVP subpopulations. Given the high heterogeneity of EVPs, different subtypes may possess distinct characteristics such as transmission efficiency and duration of action, potentially participating in various regulatory pathways, thus warranting further comprehensive investigation.

In conclusion, this preliminary study provides a fresh perspective on the long‐range effects of tumors on liver metabolism through EVPs. Future translational research should emphasize the specificity of diagnosis, optimal timing for treatment, and differences in characteristics among EVP subtypes. Tumor‐drive EVPs possess significant potential to influence systemic metabolism, and with further investigation, they can play a crucial role in improving the metabolic status of cancer patients and enhancing the effectiveness of cancer treatment.

## AUTHOR CONTRIBUTION

X. S. analyzed the literature, wrote the manuscript, and drafted the figure. X. S., P. Z., and S. D. conceived the idea. P. Z. and S. D. reviewed and revised the manuscript. All authors have read and approved the final manuscript.

## CONFLICT OF INTEREST

The authors declare that they have no competing interests.

## ETHICS STATEMENT

Not applicable.

## Data Availability

Not applicable.
